# Angiotensin II-induced upregulation of SGLT1 and 2 contributes to human microparticle‐stimulated endothelial senescence and dysfunction: protective effect of gliflozins

**DOI:** 10.1186/s12933-021-01252-3

**Published:** 2021-03-16

**Authors:** Sin-Hee Park, Eugenia Belcastro, Hira Hasan, Kensuke Matsushita, Benjamin Marchandot, Malak Abbas, Florence Toti, Cyril Auger, Laurence Jesel, Patrick Ohlmann, Olivier Morel, Valérie B. Schini-Kerth

**Affiliations:** 1 Regenerative Nanomedicine, Faculty of Pharmacy, UMR 1260, INSERM (French National Institute of Health and Medical Research), University of Strasbourg, 67000 Strasbourg, France; 2 Service de Cardiologie, Hôpitaux Universitaires de Strasbourg, 67000 Strasbourg, France

**Keywords:** Endothelial senescence and dysfunction, Angiotensin II, Circulating microparticles, SGLT1, SGLT2, Empagliflozin

## Abstract

**Background:**

Sodium-glucose cotransporter 2 (SGLT2) inhibitors reduced cardiovascular risk in type 2 diabetes patients independently of glycemic control. Although angiotensin II (Ang II) and blood-derived microparticles are major mediators of cardiovascular disease, their impact on SGLT1 and 2 expression and function in endothelial cells (ECs) and isolated arteries remains unclear.

**Methods:**

ECs were isolated from porcine coronary arteries, and arterial segments from rats. The protein expression level was assessed by Western blot analysis and immunofluorescence staining, mRNA levels by RT-PCR, oxidative stress using dihydroethidium, nitric oxide using DAF-FM diacetate, senescence by senescence-associated beta-galactosidase activity, and platelet aggregation by aggregometer. Microparticles were collected from blood of patients with coronary artery disease (CAD-MPs).

**Results:**

Ang II up-regulated SGLT1 and 2 protein levels in ECs, and caused a sustained extracellular glucose- and Na^+^-dependent pro-oxidant response that was inhibited by the NADPH oxidase inhibitor VAS-2780, the AT1R antagonist losartan, sotagliflozin (Sota, SGLT1 and SGLT2 inhibitor), and empagliflozin (Empa, SGLT2 inhibitor). Ang II increased senescence-associated beta-galactosidase activity and markers, VCAM-1, MCP-1, tissue factor, ACE, and AT1R, and down-regulated eNOS and NO formation, which were inhibited by Sota and Empa. Increased SGLT1 and SGLT2 protein levels were observed in the rat aortic arch, and Ang II- and eNOS inhibitor-treated thoracic aorta segments, and were associated with enhanced levels of oxidative stress and prevented by VAS-2780, losartan, Sota and Empa. CAD-MPs promoted increased levels of SGLT1, SGLT2 and VCAM-1, and decreased eNOS and NO formation in ECs, which were inhibited by VAS-2780, losartan, Sota and Empa.

**Conclusions:**

Ang II up-regulates SGLT1 and 2 protein expression in ECs and arterial segments to promote sustained oxidative stress, senescence and dysfunction. Such a sequence contributes to CAD-MPs-induced endothelial dysfunction. Since AT1R/NADPH oxidase/SGLT1 and 2 pathways promote endothelial dysfunction, inhibition of SGLT1 and/or 2 appears as an attractive strategy to enhance the protective endothelial function.

## Background

Endothelial dysfunction characterized by impaired endothelial cellular function including blunted nitric oxide (NO) formation, oxidative stress, endothelial senescence, and increased microparticles (MPs) shedding, is an early hallmark of the development of cardiovascular diseases that is initially affecting highly localized arterial sites which are exposed to disturbed flow and low shear such as bifurcations and curvatures [1, 2]. Endothelial dysfunction is an independent predictor of atherothrombotic events in coronary artery disease (CAD) patients and has lately been proposed as a key determinant of outcome in heart failure with preserved ejection fraction (HFpEF) [3–5]. Plasma membrane-derived circulating MPs have emerged as a surrogate biomarker and effector of endothelial dysfunction and cardiovascular risk [6], including heart failure [7–9] and behave as a biological transcellular signal delivery system promoting vasoconstriction, vascular oxidative stress, fibrosis and remodeling, and also proinflammatory responses [10]. MPs can also contribute to endothelial senescence and dysfunction especially by modulating the NO/reactive oxygen species (ROS) balance in favor of oxidative stress, which promotes procoagulant and proinflammatory responses [11, 12]. Indeed, exposure of endothelial cells (ECs) to circulating MPs from acute coronary syndrome patients induced premature endothelial senescence and thrombogenicity through activation of the Ang II/AT1R/NADPH oxidase pathway [13]. Such findings are in good agreement with observations indicating that the angiotensin system contributes to the induction of endothelial dysfunction in the inner curvature of the aortic arch [14], in experimental models of atherosclerosis and aging, hypertension, diabetes and in patients at high cardiovascular risk [15–17]. ECs express angiotensin-converting enzyme (ACE) that stimulates the conversion of angiotensin I (Ang I) into the biologically active Ang II [18], which, in turn, causes NADPH oxidase-mediated oxidative stress and promotes vasoconstriction, endothelial senescence and dysfunction, and vascular and cardiac remodeling [19, 20].

Recent findings have emphasized the potential role of sodium-glucose cotransporters (SGLTs), which transport glucose across the plasma membrane *via* a symport mechanism and the concomitant transfer of sodium in the development of cardiovascular disease. Several clinical trials have indicated cardiovascular beneficial effects of SGLT2 inhibitors by lowering mortality from cardiovascular causes and hospitalization for heart failure in type 2 diabetic mellitus patients (T2DM) with established cardiovascular diseases [21–23] and these effects appeared to be independent of glycemic control [24, 25]. Several potential mechanisms have been suggested to contribute to their beneficial effects including a reduction in blood pressure, arterial stiffness and albuminuria, induction of natriuresis and diuresis, improvement of the lipid profile, myocardial energetics by increasing oxidation of ketone bodies and of visceral adiposity, and weight loss [26, 27]. The fact that high glucose caused the redox-sensitive upregulation of SGLT1 and 2 through the local angiotensin system promoting endothelial senescence [28] suggests also a beneficial effect on the endothelial function. Despite of the remarkable cardiovascular benefits, the expression of SGLT1 and SGLT2 on ECs remains poorly studied as well as their role in the control of the endothelial function.

Therefore, the present study examined whether Ang II and CAD-MPs known to activate the local angiotensin system induce SGLT1 and 2 expression in ECs to promote premature senescence and dysfunction. In addition, the possibility that SGLT1 and 2 contribute in a feedforward manner to sustain the pro-oxidant response and the deleterious effects of both Ang II and CAD-MPs on ECs was evaluated. Moreover to assess the physiological relevance, experiments have investigated the expression level of SGLT1 and 2 at an arterial site prematurely affected by endothelial dysfunction and exposed to disturbed flow and low shear stress (aortic arch) and an arterial site at low risk exposed to laminar flow and high shear stress (thoracic aorta), and also in thoracic aorta segments exposed to either Ang II or following inhibition of the endothelial formation of NO in adult rats.

## **Materials and methods**

### Materials

Empagliflozin was provided by Boehringer Ingelheim Pharma GmbH & Co KG (Biberach an der Riss, Germany) and sotagliflozin was from CliniSciences (Nanterre, France). All other chemicals were from Sigma-Aldrich (Sigma-Aldrich Chimie SARL, St Quentin Fallavier, France) unless otherwise specified.

### Animals and ex vivo treatment of rat aorta

Male Wistar rats (10 week-old) were obtained from Janvier labs (Le Genest St Isle, France). Only male rats were studied because of their more stable hormonal profile than female rats. After 1 week, rats were euthanized by injection of an overdose of ketamine and xylazine (120 and 20 mg/kg, respectively, i.p.). The aortic arch and thoracic aorta were isolated and cleaned of connective tissue. The thoracic aorta was cut into 6 segments (3–4 mm in length). Segments of aortic arch and thoracic aorta were incubated in MCDB 131 medium (Invitrogen) supplemented with fungizone (2.5 µg/ml), penicillin (100 U/ml), streptomycin (100 µg/ml), L-glutamine (2 mM, all from Lonza, Levallois-Perret, France) for 15 h. In the case of the thoracic aorta, segments were incubated in the absence or presence of either a NADPH oxidase inhibitor (VAS-2870, 1 µM), an AT1R antagonist (losartan, 1 µM), a dual SGLT1 and SGLT2 inhibitor (sotagliflozin, 100 nM) or a selective SGLT2 inhibitor (empagliflozin, 100 nM) for 30 min before the addition of Ang II (100 nM) or a NO synthase inhibitor (N^ω^-nitro-L-arginine, L-NA, 300 µM) for 15 h. Thereafter, segments were washed with phosphate-buffered saline solution (PBS) without calcium before being snap frozen or embedded into FSC22 Blue Frozen Section Compound (Leica Biosystems, France) and then frozen in liquid nitrogen, and stored at − 80 °C.

### Patients and isolation of circulating microparticles

The Institutional Review Board has approved the study and all participants gave informed consent. Twenty-six patients with CAD (between 50 and 88 years old) were enrolled at the University Hospital of Strasbourg, France. The extent of CAD was characterized by coronary angiography. Patients with a history of chronic inflammatory disorders or atrial fibrillation were excluded. Clinical characteristics of patients are given in the Additional file 1: Table S1.

Blood samples collected by arterial puncture into tubes containing 129 mM sodium citrate were processed within 1 h 30 min. Platelet-poor plasma (PPP) samples containing circulating MPs were acquired by double centrifugations at room temperature and immediately stored at − 80 °C until use as previously described [29]. For ex vivo experiments, PPP samples from individual CAD patients were thawed and centrifuged twice at 14,000*g* for 1 h at 4 °C. The MPs pellets after centrifugation were concentrated in calcium and magnesium-free Hank balanced salt solution (HBSS) before the addition to ECs. To determine the effects of modulators, two series of washed MPs were produced from pooled PPP samples of 6 and 5 CAD patients. Briefly, 50 ml of PPP samples were subjected to a double centrifugation step (14,000*g*, 1 h, 4 °C) and the final pellet was resuspended in 1.2 ml of HBSS.

Two MP isolation procedures were performed, based on capture by 2 types of biotinylated ligands: annexin-V or specific antibodies. Ligands were separately insolubilized onto streptavidin-coated microtitration plates before incubation with PPP. This capture system allows the extensive washing ensuring specific MP binding, lipoproteins are not captured. Each capture procedure shows a specific advantage for the assessment of circulating MPs in clinical subsets. Annexin-V probes PhtdSer accessibility at the MPs surface, whereas mAbs target plasma membrane proteins. Because PhtdSer is a ubiquitous feature of MPs, the quantity of MPs captured on annexin-V provides information on the total amount of circulating procoagulant MPs, regardless of their cellular origin. The quantity of MPs captured onto specific antibodies identifies the shedding cell type and may give additional indication on the cell response to a specific vascular stress, according to the membrane antigens eventually sorted out in MPs. Identical batches of mAb to various cell types or phenotypes were used throughout all assays: anti-CD11a for leukocytes, anti-CD31 and CD105 for ECs, anti-GPIb for platelets. Background values in the quantification of MPs were obtained with corresponding irrelevant immunoglobulin (Ig) Gs and subtracted. The concentration of MPs was measured by prothrombinase assay using a microplate reader set in kinetics software and referred to as nM phosphatidylserine equivalent (nM PhtdSer eq). The phosphatidylserine content of MPs captured onto annexin-V were detected at 405 nm using a chromogenic substrate for thrombin. No direct comparison between capture by annexin-V and antibodies can be done because affinities for the respective ligands and incubation times are different. Variations in measurements are routinely less than 10 % (identical PPP sample assayed on 15 separate occasions), regardless of the pathologic issue and of the capture system (annexin-V or antibodies) [30]. Finally, analysis of MPs size was performed using qNano Gold system as recommended.

### Cell culture

Porcine hearts were obtained from the local slaughterhouse (SOCOPA, Holtzheim, France) and ECs were isolated from porcine left circumflex coronary arteries as described previously [31]. Briefly, porcine left circumflex coronary arteries were dissected and cleaned of connective tissues. After washing with PBS without calcium to remove remaining blood, ECs were isolated by type I collagenase (Invitrogen) treatment at 1 mg/ml for 15 min at 37 °C and cultured in a T25 flask containing MCDB 131 medium supplemented with 15 % fetal calf serum, fungizone (2.5 µg/ml), penicillin (100 U/ml), streptomycin (100 µg/ml), L-glutamine (2 mM) and grown to 80–90 % confluence for 48–72 h (passage 0). All experiments were performed with cultured ECs at passage 1, which were treated 15 h after passaging. ECs were exposed to serum-free culture medium for 2 h before the addition of Ang II or CAD-MPs. In some experiments, ECs were pretreated with a pharmacological modulator for 30 min before the addition of Ang II or CAD-MPs. In experiments with CAD-MPs, ECs were incubated with CAD-MPs at 10 nM PhtdSer eq for 48 h.

### Western blot analysis

After treatment, ECs washed with cold PBS and frozen segments of the aortic arch and thoracic aorta were homogenized in extraction buffer (composition in mM: Tris/HCl 20 (pH 7.5), NaCl 150, Na_3_VO_4_ 1, Na_4_P_2_O_7_ 10, NaF 20, okadaic acid 0.01, 1 % Triton X-100 and protease inhibitor cocktail (Complete Mini, Roche)). Total proteins (15 µg) were separated on 8 or 12 % SDS polyacrylamide gels and transferred electrophoretically onto nitrocellulose membrane (GE Healthcare Life Sciences). After blocking with 5 % bovine serum albumin in Tris-buffered saline containing 0.1 % Tween 20 for 1 h at room temperature, membranes were incubated with a primary antibody against either rabbit polyclonal anti-SGLT1 (for porcine; 1:1,000, Abcam, ab14685, for rat; 1:1000; Santa Cruz Biotechnology; sc-98974), rabbit polyclonal anti-SGLT2 (for porcine; 1:1,000, Abcam, ab37296, for rat; 1:1,000; Santa Cruz Biotechnology; sc-98975), rabbit polyclonal anti-angiotensin-converting enzyme (ACE, 1:1,000, Abbiotec, 250450), rabbit polyclonal anti-angiotensin type 1 receptor (AT1R, 1:1,000, Abcam, ab124505), mouse monoclonal anti-eNOS (1:5,000, BD Transduction Laboratories, 610297), rabbit monoclonal anti-VCAM-1 (1:10,000, Abcam, ab134047), rabbit polyclonal anti-MCP-1 (1:1,000, Abcam, ab25124), rabbit polyclonal anti-tissue factor (TF, 1:1,000, Sekisui Diagnostics, 4509), mouse monoclonal anti-KLF4 (1:1,000, Santa Cruz Biotechnology, sc-166238), mouse monoclonal anti-α-Actin (1:1,000, Santa Cruz Biotechnology, sc-32251) or mouse monoclonal anti-β-tubulin (1:20,000, Sigma-Aldrich, T7816) overnight at 4 °C. After washing, membranes were incubated with the secondary antibody (peroxidase-labeled anti-rabbit or anti-mouse immunoglobulin G, 1:10,000, Cell Signaling Technology, #7074, #7076, respectively) for 1 h at room temperature. The immunoreactive bands were developed by enhanced chemiluminescence (ECL, Amersham) using ImageQuant LAS 4000 (GE Healthcare).

### Immunofluorescence staining

ECs were cultured on 8-well Lab-Tek® chambers and exposed to either H_2_O_2_ (100 µM) or Ang II (100 nM) for 24 h. Cells were fixed during 30 min with 4 % (w/v) paraformaldehyde and then incubated with blocking/permeabilizing buffer (PBS containing 1 % BSA (w/v) and 0.5 % Triton X-100 (w/v)) for 30 min at room temperature. After buffer removal, cells were incubated with 1:100 dilution of either rabbit anti-SGLT1 or SGLT2 for 1 h at 4 °C. After washing 3 times with PBS, they were further incubated with a 1:250 dilution of a polyclonal goat anti-rabbit immunoglobulin G coupled to CF 633 (Alexa Fluor 633 conjugate, Invitrogen) for 1 h at room temperature in the dark. After washing 3 times with PBS, cells were incubated with 1 mg/ml 4’,6-diamidino-2’-phenylindole dihydrochloride (DAPI, Thermo Fisher) during 3 min at room temperature, in order to counterstain nuclei. After disassembling, slides were mounted with fluorescent mounting medium. Images were acquired using a Leica TCS SPE confocal microscope.

### Cellular and in situ level of oxidative stress

ECs were cultured on 8-well Lab-Tek® chambers and pretreated with either an antioxidant (N-acetyl cysteine, 1 mM) for 2 h, VAS-2870 (1 µM), a cyclooxygenase inhibitor (indomethacin, 30 µM), a mixture of mitochondrial respiratory chain inhibitors (myxothiazol, KCN and rotenone, 0.5, 1, 1 µM, respectively), losartan (1 µM), sotagliflozin (100 nM) or empagliflozin (100 nM) for 30 min before the addition of Ang II for 30 min or 24 h. To determine the contribution of glucose and sodium, ECs after a 24-h treatment period with Ang II were exposed to different concentrations of glucose (0, 0.344, 1.375, 5.5, 10, 15, 20 and 25 mM) for 1 h in either sodium-containing buffer (mM: NaCl 140, KCl 5, CaCl_2_ 2.5, MgSO_4_ 1, KH_2_PO_4_ 1, and HEPES 10, pH 7.4) or sodium-free buffer with NaCl replaced by N-methyl-D-glucamine-Cl. To evaluate the role of glucose metabolism, a non-metabolizable glucose analogue, methyl α-D-glucopyranoside (AMG, 25 mM) was tested and mannitol (25 mM) was used to rule out an osmotic effect. In some experiments, ECs were incubated with a Na^+^/H^+^ exchanger (NHE)-1 inhibitor (cariporide, 10 µM), a Na^+^/Ca^2+^ exchanger (NCX) inhibitor (KB-R7943, 10 µM) or a Na^+^/K^+^-ATPase (NKA) inhibitor (ouabain, 10 nM). For *in situ* experiments, aorta cryosections (25 µm) were incubated with either sotagliflozin (100 nM) or empagliflozin (100 nM) for 30 min at 37 °C. Thereafter, cells and aorta sections were exposed to dihydroethidium (5 µM), a redox-sensitive fluorescent dye for 30 min at 37 °C in the dark. After washing 3 times with PBS, cells and aorta sections were mounted with fluorescent mounting medium. Images were acquired using a Leica TCS SPE confocal microscope.

### Transfection of siRNA to ECs

ECs were transfected with siRNA (40 nM) targeting SGLT2 (Eurogentec; SGLT2 siRNA sense GCCUCAAUCUUUAACAGCA, antisense UGCUGUUAAAGAUUGAGGC), negative control siRNA sense UCACCAUGAUCUACACUGU, antisense ACAGUGUAGAUCAUGGUGA) for 6 h before the addition of Ang II (100 nM) for 24 h. Transfections were conducted using Lipofectamine 3000 (Invitrogen) according to the manufacturer’s instructions.

### Determination of senescence‐associated β-galactosidase (SA-β-gal) activity

Fluorescence-based SA-β-gal activity was determined in ECs using 5-dodecanoylaminofluorescein di-β-D-galactopyranoside (C_12_FDG), a membrane-permeable fluorogenic substrate of β-galactosidase, by flow cytometry as described previously [32]. ECs were pretreated with either VAS-2870 (1 µM), losartan (1 µM), sotagliflozin (100 nM), empagliflozin (100 nM), cariporide (10 µM) or KB-R7943 (10 µM) for 30 min before the addition of Ang II (100 nM) for 24 h. ECs were exposed to chloroquine (300 µM), a lysosomal inhibitory drug, for 1 h before the addition of C_12_FDG (33 µM) for 1 h. After washing twice with PBS, ECs were harvested by trypsinization and centrifuged at 10,000 rpm for 10 min at 4 °C followed by resuspension in ice-cold PBS. The relative SA-β-gal activity was estimated using the MFI of the population determined by BD FACSCelesta flow cytometer.

### mRNA expression by quantitative RT-PCR

Total RNA was isolated from ECs using NucleoSpin® RNA Plus kit (Machery-Nagel). RNA isolated from ECs (500 ng) was used to synthesize cDNA using the Maxima H Minus First Strand cDNA Synthesis Kit, with dsDNase (Thermo Fisher). RT-qPCR was performed with SYBR® Green Master Mix (Applied Biosystems) using a StepOnePlus Real-Time PCR System (Applied Biosystems). Primer sequences are shown in Additional file 1: Table S3. 18s, Hprt and Gusb were used as housekeeping genes. Relative quantitation was determined by standard 2^(−ΔΔCT)^ calculations.

## Cellular level of NO

ECs were cultured on 8-well Lab-Tek® chambers and treated with either sotagliflozin (100 nM) or empagliflozin (100 nM) for 30 min before the addition of Ang II for 24 h or CAD-MPs for 48 h. ECs were exposed to DAF-FM diacetate (4-amino-5-methylamino-2’,7’-difluororescein diacetate, 1 µM), a NO-sensitive fluorescent dye, for 20 min at 37 °C in the dark. The formation of NO was induced by the exposure of ECs to bradykinin (100 nM) for 15 min. After washing 3 times with PBS, cells were mounted with fluorescent mounting medium. Images were acquired using a Leica TCS SPE confocal microscope.

## Determination of platelet aggregation

Platelets isolated and suspended in Tyrode buffer at 310,000 platelets/µl from healthy human blood were obtained from the Etablissement Français du Sang-Alsace, Strasbourg. Suspensions of platelets (450 µl) were incubated into a cuvette with stirring at 37 °C in an aggregometer (Chronolog 490, Diagnostica Stago SAS, Asnière sur Seine, France). ECs were cultured on Cytodex 3 microcarrier beads and pretreated with either sotagliflozin (100 nM) or empagliflozin (100 nM) for 30 min before the addition of Ang II for 24 h. ECs on Cytodex 3 beads (about 500 cells) were added to suspensions of platelets for 1 min before the addition of bradykinin (100 nM) to stimulate the endothelial formation of NO for 1 min. Thereafter, a thromboxane A_2_ analog (U46619, 0.3 µM) was added to induce platelet aggregation.

### Statistical analysis

Values are expressed as means ± SEM. Statistical analysis was assessed by one-way analysis of variance followed by Tukey’s multiple comparison *post hoc* test using GraphPad Prism (Version 7). Group differences were considered statistically significant at *P* < 0.05.

## Results

### Ang II up‐regulates the expression of SGLT1 and 2 in ECs

**Fig. 1:**
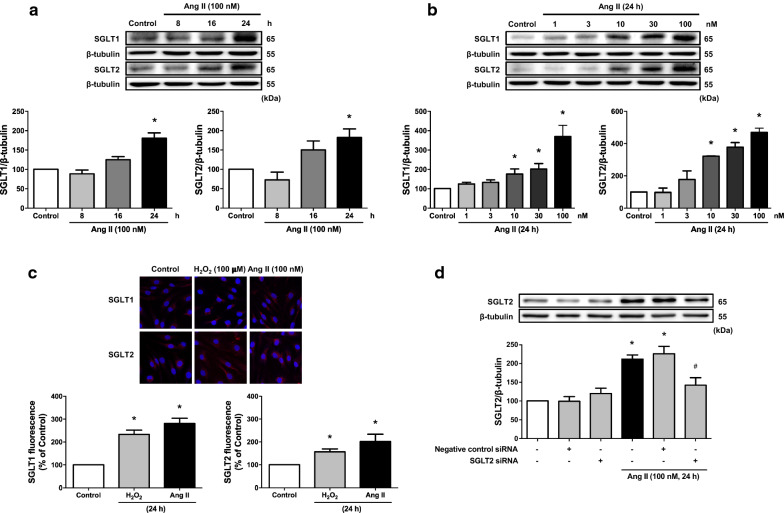
Ang II up-regulates in a time- and concentration-dependent manner the protein level of SGLT1 and SGLT2 in ECs as assessed by Western blot (**a**, **b**) and immunofluorescence staining (**c**). SGLT1 and SGLT2 protein staining appears in red and nuclei are stained with DAPI (blue). Exposure of ECs to SGLT2 siRNA (40 nM) for 6 h prevented the stimulatory effect of Ang II on SGLT2 (**d**). Results are shown as representative immunoblots and micrography of immunofluorescence staining (upper and left panels) and corresponding cumulative data (lower and right panels). Data are expressed as mean ± SEM of n = 3. ^*^P < 0.05 vs. control and ^#^*P* < 0.05 vs. Ang II

Low signals of SGLT1 and SGLT2 proteins were observed in control ECs (Fig. 1a). These signals increased in response to Ang II in a time-dependent manner reaching both about 180 % after a 24-h stimulatory period (Fig. 1a). The response to Ang II at 24 h was concentration-dependent with significant increased levels at concentrations greater than 10 nM, and reaching about 370 and 470 % at 100 nM, respectively (Fig. 1b). Higher SGLT1 and 2 fluorescence signals were also observed in ECs in response to Ang II and to H_2_O_2_ (Fig. 1c). The stimulatory effect of Ang II on SGLT2 protein levels was not observed following pre-treatment of ECs with a SGLT2 siRNA or with an inactive sequence (Fig. 1d).

### Role of SGLT1 and 2 in the Ang II-induced pro-oxidant response in ECs

**Fig. 2:**
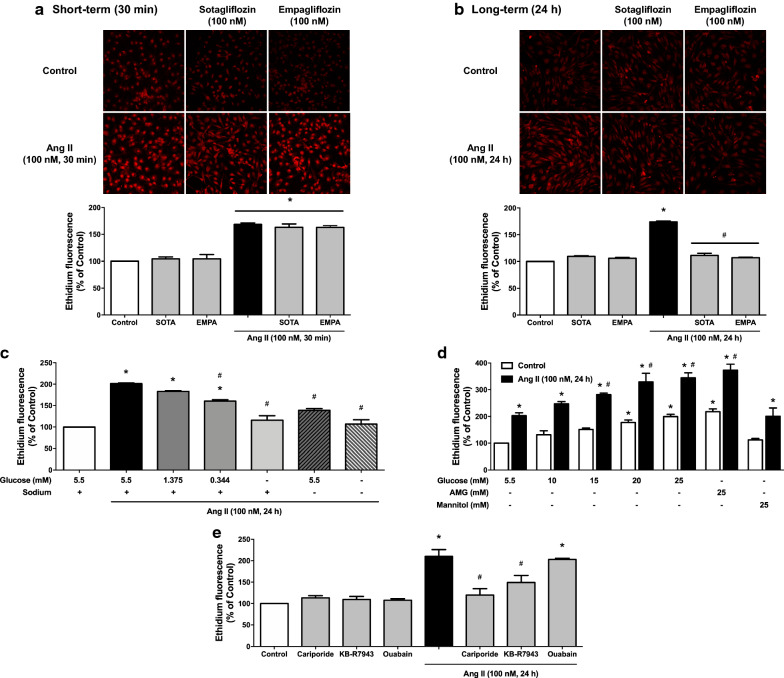
The sustained Ang II-induced formation of ROS in ECs is sensitive to a dual SGLT1 and SGLT2 inhibitor, sotagliflozin, and a selective SGLT2 inhibitor, empagliflozin. ECs are incubated with either (**a**, **b**) sotagliflozin (SOTA, 100 nM) or empagliflozin (EMPA, 100 nM) for 30 min before the addition of Ang II for either 30 min (**a**) or 24 h (**b**). For characterization of the role of SGLT1 and 2 in the pro-oxidant response to Ang II, ECs are incubated with Ang II for 24 h before being exposed to (**c**) the indicated glucose concentrations for 1 h in the presence or absence of sodium, (**d**) the indicated glucose concentrations, methyl α-D-glucopyranoside (AMG, a non-metabolizable glucose analogue), or mannitol, and (**e**) cariporide (a NHE-1 inhibitor, 10 µM), KB-R7943 (a NCX inhibitor, 10 µM), or ouabain (a NKA inhibitor, 10 nM) for 1 h, and the subsequent determination of dihydroethidium staining by confocal microscope. Results are shown as representative micrography of dihydroethidium staining (upper panels) and corresponding cumulative data (lower panels). Data are expressed as mean ± SEM of n = 3. ^*^P < 0.05 vs. control and ^#^*P* < 0.05 vs. Ang II

Since Ang II is a potent inducer of the generation of ROS in ECs [33], the possibility that SGLT1 and 2 may contribute to the pro-oxidant response was evaluated using dihydroethidium. Exposure of ECs to Ang II increased the level of ethidium fluorescence after 30 min and the stimulatory effect persisted up to 24 h (Fig. 2 and Additional file 1: Figure S1A and B). Both the short and sustained pro-oxidant responses to Ang II were abolished by the antioxidant N-acetyl cysteine, and the AT1R antagonist losartan (Additional file 1: Figure S1A and B). The characterization of the Ang II-triggered formation of ROS has indicated that the NADPH oxidase inhibitor, VAS-2870, the cyclooxygenase inhibitor, indomethacin and inhibitors of the mitochondrial respiratory chain (combination of myxothiazol, KCN and rotenone) all significantly inhibited both short-term and long-term pro-oxidant responses, indicating the involvement of several sources including NADPH oxidase, cyclooxygenases and the mitochondrial respiratory chain (Additional file 1: Figure S1A and B).

Next the possibly that SGLT1 and 2 contribute to the pro-oxidant response to Ang II in ECs was determined using a dual SGLT1 and 2 inhibitor, sotagliflozin and a selective SGLT2 inhibitor, empagliflozin. The 24-h but not the 30-min pro-oxidant response to Ang II was abolished by sotagliflozin and empagliflozin (Fig. 2a, b), indicating that although SGLT1 and 2 do not contribute to the early pro-oxidant response, they have a crucial role in perpetuating the pro-oxidant response. Sotagliflozin and empagliflozin alone did not affect the low basal formation of ROS after 30 min and 24 h in ECs (Fig. 2a, b).

To further characterize the role of SGLT1 and 2 in the pro-oxidant response to Ang II, the role of extracellular glucose and Na^+^ was assessed. The pro-oxidant response of Ang II at 24 h was significantly reduced by decreasing the extracellular concentration of glucose and also by replacing extracellular Na^+^ by N-methyl-D-glucamine, and abolished in the absence of both extracellular glucose and Na^+^ (Fig. 2c), indicating a key role of both extracellular glucose and Na^+^ possibly subsequent to their entry *via* SGLT1 and 2.

In addition, exposure of ECs to increasing concentrations of glucose from 5.5 to 25 mM for 24 h induced a pro-oxidant response at concentrations greater than 20 mM (Fig. 2d). Moreover, the combined treatment of ECs with Ang II and high glucose resulted in an additive pro-oxidant response (Fig. 2d). A similar potentiating effect of the pro-oxidant response to Ang II was also observed in response to a non-metabolizable glucose analogue, AMG, which has been shown to enter cells *via* SGLTs (Fig. 2d) [34, 35]. In contrast, no such effect was observed with mannitol ruling out an osmotic effect (Fig. 2d). Thus, Ang II and high glucose act together to promote an excessive level of oxidative stress that is independent of glucose metabolism. Moreover, the sustained pro-oxidant response to Ang II was markedly inhibited by cariporide (Na^+^/H^+^ exchanger-1 inhibitor) and KB-R7943 (Na^+^/Ca^2+^ exchanger inhibitor), but not affected by ouabain (Na^+^/K^+^-ATPase inhibitor) suggesting the involvement of NHE-1 and NCX, besides SGLT1 and 2 (Fig. 2e).

### SGLT1 and 2 act in a feedforward manner to sustain the stimulatory effect of the Ang II/AT1R/NADPH oxidase/ROS pathway on SGLT1 and 2 expression in ECs

**Fig. 3:**
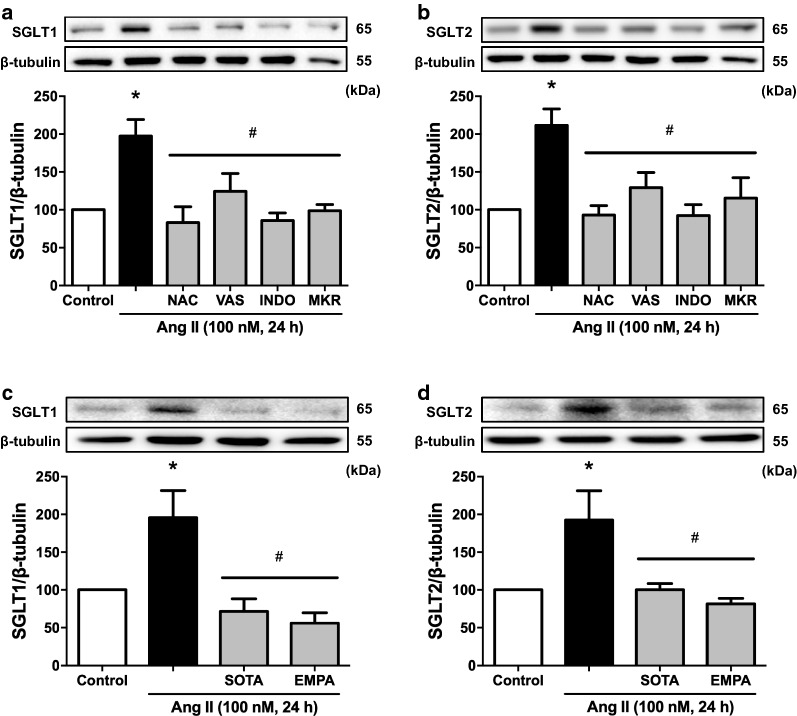
Ang II causes a redox-sensitive up-regulation of SGLT1 and 2 promoting their own expression in ECs. ECs are incubated with either (**a**, **b**) *N*-acetyl cysteine (NAC, an antioxidant, 1 mM) for 2 h, VAS-2870 (VAS, a NADPH oxidase inhibitor, 1 µM), indomethacin (INDO, a cyclooxygenase inhibitor, 30 µM) or myxothiazol (0.5 µM) + KCN (1 µM) + rotenone (1 µM; MKR, mitochondrial respiratory chain inhibitors) for 30 min, and (**c**, **d**) sotagliflozin (SOTA, 100 nM) or empagliflozin (EMPA, 100 nM) for 30 min before the addition of Ang II and the subsequent assessment of the expression level of SGLT1 and SGLT2 by Western blot analysis. Results are shown as representative immunoblots (upper panels) and corresponding cumulative data (lower panels). Data are expressed as mean ± SEM of n = 3. ^*^P < 0.05 vs. control and ^#^*P* < 0.05 vs. Ang II

Since H_2_O_2_ induced the expression of SGLT1 and 2 in ECs [28], the role of ROS in the Ang II-induced expression of SGLT1 and 2 was evaluated. All inhibitors of the pro-oxidant response to Ang II (N-acetyl cysteine, VAS-2870, indomethacin and the mitochondrial respiratory chain inhibitors) abolished the stimulatory effect of Ang II on SGLT1 and 2 protein expression levels (Fig. 3a, b) demonstrating a crucial redox-sensitive mechanism. Moreover, the fact that sotagliflozin and empagliflozin abolished the Ang II-induced up-regulation of both SGLT1 and 2 (Fig. 3c, d) suggests that activation of SGLT1 and 2 contributes to promote their own expression.

### Role of SGLT1 and 2 in Ang II-induced senescence and dysfunction in ECs

**Fig. 4:**
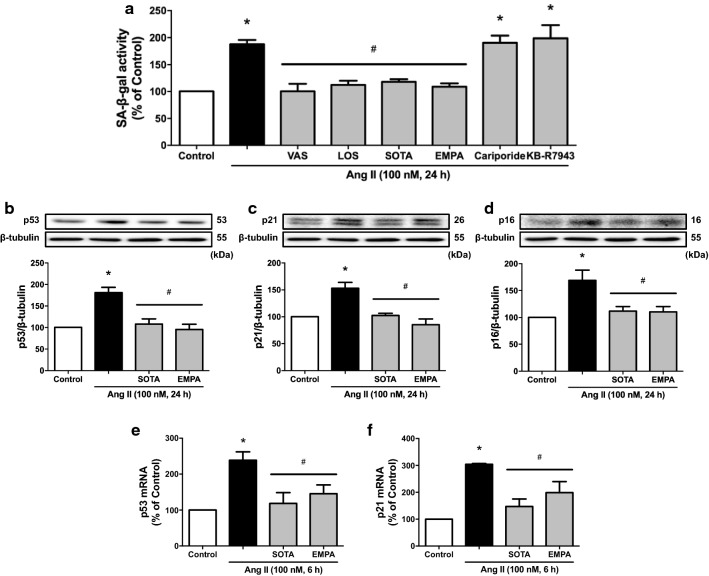
Ang II-induced endothelial senescence is dependent on the AT1R/NADPH oxidase/SGLT1 and 2 pathways. ECs are incubated with either VAS-2870 (VAS, 1 µM), losartan (LOS, an AT1R antagonist, 1 µM), sotagliflozin (SOTA, 100 nM), empagliflozin (EMPA, 100 nM), cariporide (10 µM) or KB-R7943 (10 µM) for 30 min before the addition of Ang II, and the subsequent determination of (**a**) SA-β-gal activity, (**b**–**d**) the expression level of p53, p21 and p16 protein after a 24-h incubation period by Western blot analysis, and (**e**, **f**) the expression level of p53 and p21 mRNA after a 6-h incubation period by RT-PCR. Results are shown as representative immunoblots (upper panels) and corresponding cumulative data (lower panels). Data are expressed as mean ± SEM of n = 3. ^*^P < 0.05 vs. control and ^#^*P* < 0.05 vs. Ang II

Since Ang II is a potent redox-sensitive inducer of senescence in ECs [36], the role of SGLT1 and 2 was evaluated using SA-β-gal activity. Ang II increased SA-β-gal activity, which was significantly inhibited by losartan and VAS-2870, and by sotagliflozin and empagliflozin, but not by cariporide and KB-R7943 (Fig. 4a), indicating the involvement of the AT1R/NADPH oxidase/SGLT1 and 2 pathways in the pro-senescence response. Consistent with the SA-β-gal activity, Ang II upregulated the expression of senescence markers p53 and p21 at both the mRNA and protein levels, and also the p16 protein level whereas the mRNA level was below detection, and all these effects were abolished by sotagliflozin and empagliflozin (Fig. 4b–f).

**Fig. 5:**
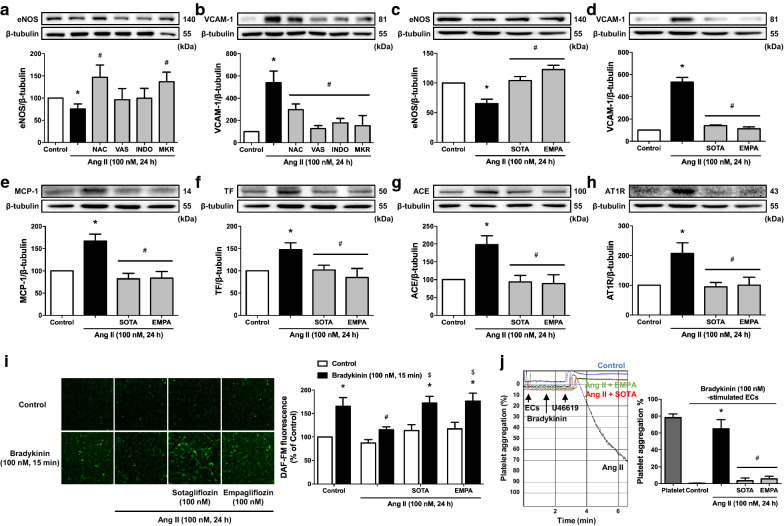
Ang II-induced pro-atherothrombotic responses in ECs are dependent on oxidative stress and SGLT1 and 2. ECs are incubated with either (**a**, **b**) NAC (1 mM) for 2 h, VAS (1 µM), INDO (30 µM) or MKR (0.5, 1, 1 µM, respectively), and (**c**–**i**) SOTA (100 nM) or EMPA (100 nM) for 30 min before the addition of Ang II and the subsequent determination of (**c**–**h**) the expression level of target proteins by Western blot analysis, (**i**) the formation of NO in response to bradykinin as assessed by DAF-FM, and (**j**) the inhibitory effect of bradykinin-stimulated ECs on platelet aggregation induced by U46619 using an aggregometer. Results are shown as representative immunoblots, micrography of DAF-FM staining and platelet aggregation traces (upper and left panels) and corresponding cumulative data (lower and right panels). Data are expressed as mean ± SEM of n = 3. ^*^P < 0.05 vs. control, and ^#^*P* < 0.05 vs. Ang II (**a–h**, **j**) and bradykinin (**i**), and ^$^*P* < 0.05 vs. Ang II + bradykinin (**i**)

Since endothelial senescence has been identified as an upstream signaling event promoting endothelial dysfunction [37], the role of SGLT1 and 2 in Ang II-induced endothelial dysfunction was evaluated. Ang II caused a down-regulation of the protein expression level of eNOS in ECs associated with a reduced bradykinin-stimulated formation of NO and inhibitory effect on platelet aggregation, and an up-regulation of VCAM-1, MCP-1 and tissue factor (Fig. 5a–f, i and j). The Ang II-induced down-regulation of eNOS was prevented significantly by N-acetyl cysteine and the mitochondrial respiratory chain inhibitors but not by VAS-2870 and indomethacin, whereas all inhibitors of the Ang II-induced pro-oxidant response inhibited the up-regulation of VCAM-1 (Fig. 5a, b). In addition, both sotagliflozin and empagliflozin prevented the Ang II-induced down-regulation of eNOS and formation of NO in response to bradykinin, and the up-regulation of VCAM-1, MCP-1 and tissue factor indicating a determinant role of SGLT1 and 2 in the induction of endothelial dysfunction (Fig. 5c–f and i). In addition, knockdown of SGLT2 expression prevented the Ang II-induced up-regulation of VCAM-1 by 63 % (data not shown). Ang II also induced an up-regulation of ACE and AT1R, which was abolished by both sotagliflozin and empagliflozin (Fig. 5g, h), suggesting that SGLT1 and 2 have a determinant role in the Ang II/AT1R/NADPH oxidase pro-oxidant stimulatory signal.

### Role of SGLT1 and 2 in arterial endothelial dysfunction: Protective effect of NO

**Fig. 6:**
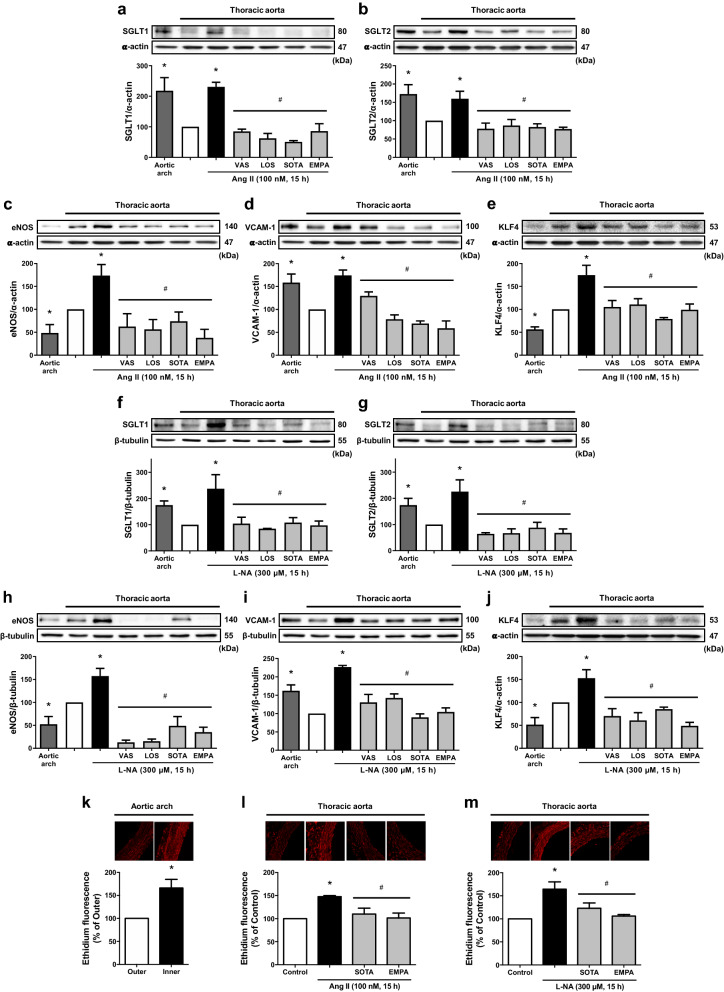
Up-regulation of SGLT1 and SGLT2 at an arterial site at high risk (aortic arch) and following stimulation of an arterial site at low risk (thoracic aorta) with either Ang II or an eNOS inhibitor. The segments of thoracic aorta are incubated either with VAS (1 µM), LOS (1 µM), SOTA (100 nM) or EMPA (100 nM) for 30 min before the addition of (**a**-**e**, **l**) Ang II (100 nM) or (**f**–**j**, **m**) N^ω^-nitro-L-arginine (L-NA, 300 µM) for 15 h, and the subsequent determination of (**a**–**J**) the expression level of target proteins by Western blot analysis, and (**k**–**m**) dihydroethidium staining by confocal microscope. Results are shown as representative immunoblots and micrography of dihydroethidium staining (upper and left panels) and corresponding cumulative data (lower and right panels). Data are expressed as mean ± SEM of n = 3–4. ^*^P < 0.05 vs. control thoracic aorta (**a**–**j**, **l**, **m**) and outer aortic arch (**k**), and ^#^*P* < 0.05 vs. Ang II-treated thoracic aorta (**a**–**e**, **l**) and L-NA-treated thoracic aorta (**f**–**j**, **m**)

To obtain physiological relevance, the expression level of SGLT1 and 2 proteins was assessed *ex vivo* at arterial sites either at high (aortic arch characterized by premature endothelial dysfunction and exposure to disturbed flow and low shear) or low risk (thoracic aorta protected by laminar flow and the high shear-induced endothelial formation of NO) [2]. An increased expression level of SGLT1 and 2 proteins was observed in the aortic arch compared to that in the thoracic aorta, and this effect was associated with an up-regulation of VCAM-1 and a down-regulation of proteins sensitive to flow including eNOS and KLF4 (Fig. 6a–j). A higher pro-oxidant level was observed in the inner curvature of the aortic arch than in the outer curvature (Fig. 6k). In addition, a 15-h exposure of thoracic aortic rings to either Ang II or an inhibitor of NO formation (L-NA) resulted in an up-regulation of SGLT1 and 2, VCAM-1 and also of eNOS and KLF4 most likely as a compensatory mechanism for the low levels of NO associated with a pro-oxidant response throughout the arterial wall (Fig. 6a–j, l and m). The Ang II- and L-NA-induced up-regulation of target proteins was prevented by losartan, VAS-2780 and also by sotagliflozin and empagliflozin, and the pro-oxidant response by sotagliflozin and empagliflozin (Fig. 6a–j, l and m). Thus, these findings indicate the involvement of AT1 receptors, NADPH oxidase-derived oxidative stress and also SGLT1 and 2, and that the endothelial formation of NO counteracts the expression of SGLT1 and 2.

### Circulating MPs from CAD patients induce expression of SGLT1 and 2 in ECs to promote endothelial dysfunction

**Fig. 7:**
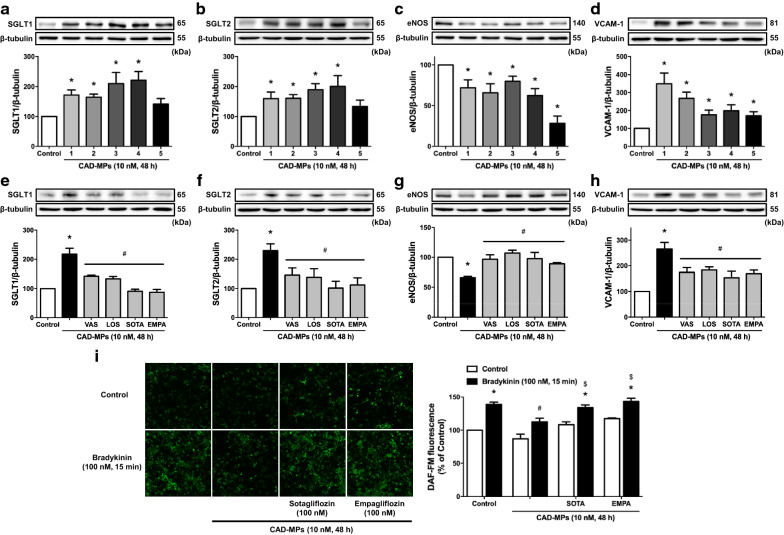
Circulating MPs from patients with coronary artery diseases (CAD) up-regulate SGLT1 and SGLT2 to promote their own expression and involve the AT1R/NADPH oxidase pathway to induce endothelial dysfunction in ECs. (**a**–**d**) ECs are exposed to CAD-MPs (10 nM PhtdSer eq) from 5 individual CAD patients. (**e**−**i**) ECs are incubated with either VAS (1 µM), LOS (1 µM), SOTA (100 nM) or EMPA (100 nM) for 30 min before the addition of CAD-MPs (10 nM PhtdSer eq) pooled from 6 (**e**–**h**) and 5 (**i**) patients with CAD for 48 h. Thereafter, (**a**–**h**) the expression level of target proteins is assessed by Western blot analysis, and **i** the formation of NO in response to bradykinin by DAF-FM. Results are shown as representative immunoblots and micrography of DAF-FM staining (upper and left panels) and corresponding cumulative data (lower and right panels). Data are expressed as mean ± SEM of n = 3. ^*^P < 0.05 vs. control, and ^#^*P* < 0.05 vs. CAD-MPs (**e**–**h**) and bradykinin (**i)**, and ^$^*P* < 0.05 vs. CAD-MPs + bradykinin (**i)**

Since circulating MPs from patients with acute coronary syndrome induced premature endothelial senescence and thrombogenicity involving the local pro-oxidant angiotensin system [13], experiments were performed to determine whether circulating MPs from CAD patients induce SGLT1 and 2 expression in ECs and, if so, to clarify their role in the induction of endothelial dysfunction. Clinical characteristics of the CAD patients are provided in the Additional file 1: Table S1. The cellular origin of patient-derived MPs was determined following their captured onto insolubilized antibodies directed either to platelets (GPIb^+^), leukocytes (CD11a^+^) or ECs (CD31^+^ and CD105^+^) and the subsequent measurement of the procoagulant MPs concentration by prothrombinase assay. The data indicated that patient-derived MPs originated from leukocytes and platelets, and also, to some extent, from ECs (Additional file 1: Table S2). The MPs size was determined using Tunable Resistive Pulse Sensing (qNano Gold system) and a NP250 nanopore (110–630 nm). The findings indicated that the diameter of MPs spanned from 181 nm (10 % percentile) to 413 nm (90 % percentile; n = 5). The value distribution peaked at a mode diameter of 195 ± 5 nm and the median value was 249 ± 4.5 nm. Of importance, no event was recorded using a NP80 nanopore (40–255 nm) indicating that the MPs preparations did not contain exosomes. The CAD-MPs from 11 out of 15 patients increased in ECs the protein expression level of both SGLT1 and 2, 15/15 patients that of VCAM-1, and 12/15 patients down-regulated that of eNOS (Fig. 7a–d, Additional file 1: Figure S2A–D). The CAD-MPs-induced down-regulation of eNOS protein level was associated with a reduced formation of NO in response to bradykinin (Fig. 7i). Moreover, the CAD-MPs-induced effect on target proteins was prevented by VAS-2870, losartan, sotagliflozin and empagliflozin, and on the bradykinin-induced formation of NO by sotagliflozin and empagliflozin (Fig. 7e–i), suggesting that the AT1R/NADPH oxidase pathway mediates the expression of SGLT1 and 2, which, in turn, contribute to the induction of endothelial dysfunction.

## Discussion

The major findings of the present study indicate that Ang II and MPs derived from CAD patients cause *via* the AT1 receptor/NADPH oxidase pathway a redox-sensitive up-regulation of the expression of SGLT1 and 2 in ECs, which, in turn, have a key role to promote ultimately endothelial senescence and dysfunction. They further indicate that SGLT1 and SGLT2 protein levels are up-regulated *ex vivo* in pathological rat arteries (i.e., aortic arch, Ang II- and eNOS inhibitor-treated thoracic aorta vs. thoracic aorta) promoting oxidative stress in the arterial wall and endothelial dysfunction most likely subsequent to the impaired endothelial formation of NO. Thus, endothelial SGLT1 and/or SGLT2 appear as novel targets for protection of the vascular system.

### Gliflozins and cardiovascular protection

Gliflozins including empagliflozin, dapagliflozin, and canagliflozin are a novel class of antidiabetic agents used for the treatment of T2DM that selectively inhibit SGLT2 to prevent glucose reabsorption in the renal proximal tubule. Cardiovascular outcome trials have shown remarkable cardioprotective effects of these selective SGLT2 inhibitors showing reduced mortality from cardiovascular causes, all-cause death and hospitalization for heart failure in T2DM patients with established cardiovascular diseases [21–23]. Since the beneficial effect on cardiovascular outcome by empagliflozin in T2DM is independent of glycemic control, the underlying mechanisms remain to be determined [24, 25]. It has been suggested that AMPK activation-mediated reduced energy depletion and inflammation contribute to the cardiovascular benefits of empagliflozin as observed 8 h after administration of lipopolysaccharide to mice [38]. Empagliflozin also attenuated the cardiotoxic effects exerted by doxorubicin on left ventricular function and remodeling in nondiabetic mice, independently of glycemic control [39]. Moreover, given the lack of reduction of macrovascular thrombotic events observed with gliflozins in clinical trials, a paradigm shift has been proposed pointing at their putative role in the improvement of coronary microvascular dysfunction which is an important determinant of prognosis in HFpEF patients [40, 41]. Recently, it has been shown that empagliflozin improved coronary microvascular function in prediabetic ob/ob^−/−^ mice [42].

 Several lines of experimental evidence suggest that gliflozins might possibly improve the pivotal protective endothelial function. Indeed, ipragliflozin improved endothelial dysfunction, restored the phosphorylation of Akt and eNOS, decreased the formation of ROS and the expression of pro-atherosclerotic factors in the abdominal aorta from streptozotocin-induced diabetic mice [43]. Moreover, empagliflozin improved endothelium-dependent relaxations in streptozotocin-induced diabetic rats [44], reduced atherosclerotic plaque formation in ApoE^−/−^ mice by improving the inflammatory response and insulin resistance [45], and improved endothelial function in an experimental model of metabolic syndrome the ZSF1 rat [46]. Recent observations have also emphasized that cardiac microvascular ECs mainly *via* endothelial-derived NO exert a direct positive effect on cardiomyocyte function, and that this effect is impaired by inflammation and prevented by empagliflozin [47, 48]. Observations also suggest that ECs senescence appears to act as a key early signal promoting endothelial dysfunction since expression of the senescent marker p53 selectively in ECs promoted endothelial dysfunction and a reduced formation of NO in isolated arteries [49]. Senescent ECs have been observed in human aortic arch [50] and coronary arteries [51] at sites overlapping atherosclerotic plaques characterized by an endothelial dysfunction. A role of SGLT1 and 2 in diabetes-related endothelial dysfunction is also supported by the fact that high glucose promoted premature senescence and pro-atherothrombotic responses in ECs and that this effect is inhibited by empagliflozin and associated with an up-regulation of the expression level of SGLT1 and 2 [28]. Consistent with those findings, a recent meta-analysis has indicated that SGLT2 inhibitors significantly improved flow-mediated dilation [52].

The angiotensin system is a major contributor to endothelial dysfunction observed prematurely at atheroprone arterial sites at risk, and also in aging- and major cardiovascular disease-related endothelial dysfunction such as coronary artery disease [14–17]. The angiotensin system promotes vascular pro-oxidant, pro-atherothrombotic and pro-senescence responses, and contributes to MPs shedding [7, 8, 53]. Indeed, circulating MPs from patients with acute coronary syndrome blunted *ex vivo* endothelium-dependent relaxations in rat aortic rings [54] and caused the induction of premature senescence in ECs *via* the Ang II-dependent NADPH oxidase-mediated formation of ROS, resulting ultimately in endothelial dysfunction [13]. Therefore, the present study examined the possibility that Ang II and CAD-MPs, activators of the local angiotensin pathway, affect the expression of SGLT1 and 2 in ECs and, if so, assessed their contribution to the induction of endothelial dysfunction.

### The AT1R/NADPH oxidase/SGLT1/2 pathway: an inducer of endothelial senescence

The present findings indicate that Ang II is a potent inducer of SGLT1 and 2 protein expression in ECs, which is mediated by oxidative stress and that this response ultimately leads to endothelial senescence and dysfunction. Of importance, these findings extend previous ones showing that the high glucose-induced expression of SGLT1 and 2 in ECs involves the local angiotensin system [28], and, thus, indicate that the stimulatory effect of Ang II is independent of hyperglycemia. The characterization of the Ang II-induced pro-oxidant response mediated *via* the AT1 receptor indicated that it is observed within 30 min and, thereafter, perpetuated for at least 24 h. In addition, both the early and sustained pro-oxidant responses involved several sources including NADPH oxidase, cyclooxygenases and the mitochondrial respiratory chain. Although sotagliflozin and empagliflozin did not affect the Ang II-induced early pro-oxidant response, both abolished the sustained response, as well as endothelial senescence and dysfunction indicating that SGLT1 and 2 appear to have a key regulatory role controlling the deleterious impact of Ang II on ECs. Moreover, the fact that sotagliflozin and empagliflozin abolished the Ang II-induced up-regulation of SGLT1 and 2 protein levels, suggests that they promote their own expression most likely by inducing uptake of Na^+^ and glucose, and, subsequently, the pro-oxidant response. The central role of SGLT1 and 2 is also supported by the fact that both sotagliflozin and empagliflozin abolished the Ang II-induced up-regulation of ACE and AT1 receptor in ECs, a major feedforward mechanism potentiating the induction of endothelial senescence and dysfunction [28].

Since SGLTs cotransport glucose and Na^+^ into the cell driven by the Na^+^ and glucose gradient, the impact of extracellular glucose and Na^+^ level on the Ang II-induced pro-oxidant response was determined. The sustained pro-oxidant response of ECs to Ang II was reduced by decreasing progressively the extracellular glucose concentration and in the absence of extracellular sodium, and also, alternatively, potentiated by increasing the extracellular concentration of glucose. Such a response was not observed with mannitol, thus excluding an osmotic effect. In addition, a similar potentiating effect as with high glucose was observed with the non-metabolizable glucose analogue, AMG, which has been shown to enter into cells primarily *via* SGLTs [34, 35]. The further characterization of the sodium pathway in the pro-oxidant response of ECs has indicated that although NHE and NCX but not the Na^+^/K^+^-ATPase are involved in the pro-oxidant response to Ang II, they do not contribute to the induction of endothelial senescence. Altogether, these findings indicate that both extracellular glucose and Na^+^ are major determinant factors setting the level of the sustained pro-oxidant response to Ang II promoting endothelial senescence most likely subsequent to their entry *via* SGLT1 and 2 in ECs. They further support the concept that SGLT1 and 2 act as glucose sensors as previously suggested in cardiomyocytes [55], hypothalamic neurons [56] and rat mesangial cells [57], and that they might contribute to hyperglycemia- and high salt intake-associated vascular complications.

Consistent with previous findings, the Ang II-induced pro-oxidant response caused the induction of endothelial senescence and dysfunction. The pathological ECs are characterized by blunted eNOS-derived NO formation, a reduced antiaggregatory effect and the up-regulation of pro-atherothrombotic makers including VCAM-1, MCP-1 and tissue factor. All of these effects were abolished by sotagliflozin and empagliflozin indicating that SGLT2 and most likely also SGLT1 are key contributors to endothelial dysfunction and the associated pro-atherothrombotic responses. *Ex vivo* investigations of rat arteries have also indicated an increased expression level of SGLT1 and 2 proteins at arterial sites at risk including the aortic arch and also in the thoracic aorta in response to either Ang II or inhibition of the eNOS-derived NO formation, which, in turn, contribute to oxidative stress and endothelial dysfunction. Moreover, the potential clinical implication of the present findings is supported by the fact that CAD-MPs were able to up-regulate the expression level of SGLT1 and 2 proteins *via* the activation of the AT1R/NADPH oxidase pathway to promote endothelial dysfunction as indicated by the down-regulation of eNOS and the bradykinin-induced formation of NO, and the up-regulation of VCAM-1, and that all these effects are abolished by sotagliflozin and empagliflozin.

## Conclusions

**Fig. 8:**
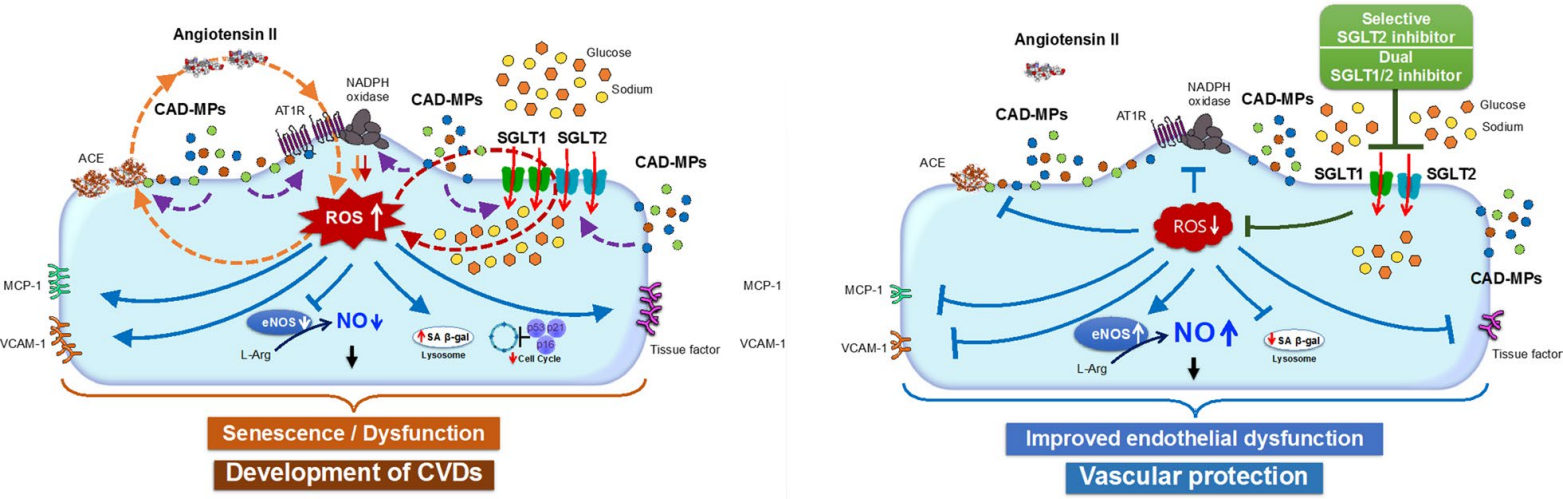
Schematic summarizing the present findings, which indicate that angiotensin II and circulating microparticles from coronary artery disease patients (CAD-MPs) *via* the local angiotensin system upregulate SGLT1 and SGLT2 expression to promote endothelial senescence and dysfunction in coronary endothelial cells. All of these effects are prevented by gliflozins

The present findings indicate that Ang II and circulating MPs from CAD patients *via* the activation of the local angiotensin system are potent inducers of SGLT1 and 2 expression to sustain the glucose- and Na^+^-dependent pro-oxidant response that ultimately leads to endothelial senescence and pro-atherothrombotic responses (Fig. 8). They further suggest that inhibition of SGLT1 and/or SGLT2 might be an attractive therapeutic strategy to protect the endothelial function, and, hence, the subsequent development of cardiovascular disease.

## Supplementary information

**Additional file 1: Table S1.** Clinical characteristics of coronary artery disease (CAD) patients. **Table S2.** Phenotype of circulating MPs in CAD patients. The concentration of circulating MPs captured onto insolubilized antibodies to platelet (GPIb^+^), leukocyte (CD11a^+^) and endothelium (CD31^+^ and CD105^+^) was measured by prothrombinase assay as nM phosphatidylserine equivalent (nM PhtdSer eq). **Table S3.** Primer sequences. Forward and reverse primer sequences of genes used for RT-qPCR showing position and size. 18S, GUSB and HPRT were used as housekeeping genes. **Figure S1.** The sustained Ang II-induced formation of ROS in ECs involves NADPH oxidase, cyclooxygenases and the mitochondrial respiratory chain. ECs are incubated with either (A, B) *N*-acetyl cysteine (NAC, an antioxidant) for 2 h, VAS-2870 (VAS, a NADPH oxidase inhibitor), indomethacin (INDO, a cyclooxygenase inhibitor), myxothiazol + KCN + rotenone (MKR, mitochondrial respiratory chain inhibitors) or losartan (LOS, an AT1R antagonist) for 30 min before the addition of Ang II for either 30 min or 24 h and the subsequent determination of dihydroethidium staining by confocal microscope. Results are shown as representative micrography of dihydroethidium staining (left panels) and corresponding cumulative data (right panels). Data are expressed as mean ± SEM of n = 3. *P < 0.05 vs control and #P < 0.05 vs Ang II. **Figure S2.** Circulating MPs from patients with CAD up-regulate SGLT1, SGLT2, and VCAM-1 and down-regulate eNOS in ECs. (A–D) ECs are exposed to CAD-MPs (10 nM PhtdSer eq) from 10 different CAD patients. Thereafter, the expression level of target proteins is assessed by Western blot analysis. Results are shown as representative immunoblots (upper panels) and corresponding cumulative data (lower panels). Data are expressed as mean ± SEM of n = 3. *P < 0.05 vs control.

## Data Availability

Not applicable.

## Abbreviations

ACE: Angiotensin-converting enzyme
AMG: Methyl α-D-glucopyranoside
Ang I: Angiotensin I
Ang II: Angiotensin II
CAD: Coronary artery disease
CAD-MPs: Circulating microparticles from coronary artery disease patients
CV: Cardiovascular
ECs: Endothelial cells
Empa: Empagliflozin
HFpEF: Heart failure with preserved ejection fraction
L-NA: N^ω^-nitro L-arginine
MPs: Microparticles
NCX: Na^+^/Ca^2+^ exchanger
NHE: Na^+^/H^+^ exchanger
NKA: Na^+^/K^+^-ATPase
NO: Nitric oxide
PBS: Phosphate-buffered saline
PPP: Platelet-poor plasma
ROS: Reactive oxygen species
SA-β-gal: Senescence-associated β-galactosidase
SGLT2: Sodium-glucose cotransporter 2
SGLTs: Sodium-glucose cotransporters
Sota: Sotagliflozin
T2DM: Type 2 diabetes mellitus patients
