# Supplementary material for: Angiotensin II-induced upregulation of SGLT1 and 2 contributes to human microparticle‐stimulated endothelial senescence and dysfunction: protective effect of gliflozins
Source: Cardiovasc Diabetol. 2021 Mar 16;20:65. doi: 10.1186/s12933-021-01252-3 (PMC7967961; doi:10.1186/s12933-021-01252-3)
Supplement: Supplementary file 1 — Additional file 1: Table S1. Clinical characteristics of coronary artery disease (CAD) patients. Table S2. Phenotype of circulating MPs in CAD patients. The concentration of circulating MPs captured onto insolubilized antibodies to platelet (GPIb+), leukocyte (CD11a+) and endothelium (CD31+ and CD105+) was measured by prothrombinase assay as nM phosphatidylserine equivalent (nM PhtdSer eq). Table S3. Primer sequences. Forward and reverse primer sequences of genes used for RT-qPCR showing position and size. 18S, GUSB and HPRT were used as housekeeping genes. Figure S1. The sustained Ang II-induced formation of ROS in ECs involves NADPH oxidase, cyclooxygenases and the mitochondrial respiratory chain. ECs are incubated with either (A, B) N-acetyl cysteine (NAC, an antioxidant) for 2 h, VAS-2870 (VAS, a NADPH oxidase inhibitor), indomethacin (INDO, a cyclooxygenase inhibitor), myxothiazol + KCN + rotenone (MKR, mitochondrial respiratory chain inhibitors) or losartan (LOS, an AT1R antagonist) for 30 min before the addition of Ang II for either 30 min or 24 h and the subsequent determination of dihydroethidium staining by confocal microscope. Results are shown as representative micrography of dihydroethidium staining (left panels) and corresponding cumulative data (right panels). Data are expressed as mean ± SEM of n = 3. *P < 0.05 vs control and #P < 0.05 vs Ang II. Figure S2. Circulating MPs from patients with CAD up-regulate SGLT1, SGLT2, and VCAM-1 and down-regulate eNOS in ECs. (A–D) ECs are exposed to CAD-MPs (10 nM PhtdSer eq) from 10 different CAD patients. Thereafter, the expression level of target proteins is assessed by Western blot analysis. Results are shown as representative immunoblots (upper panels) and corresponding cumulative data (lower panels). Data are expressed as mean ± SEM of n = 3. *P < 0.05 vs control. [file 12933_2021_1252_MOESM1_ESM.docx]

**Additional file**

**Angiotensin II-induced upregulation of SGLT1 and 2 contributes to human microparticle-stimulated endothelial senescence and dysfunction: Protective effect of gliflozins**

Sin-Hee Park^1^, Eugenia Belcastro^1^, Hira Hasan^1^, Kensuke Matsushita^2^, Benjamin Marchandot^2^, Malak Abbas^2^, Florence Toti^1^, Cyril Auger^1^, Laurence Jesel^1,2^, Patrick Ohlmann^2^, Olivier Morel^1,2*^, Valérie B. Schini-Kerth^1*^

^1^INSERM (French National Institute of Health and Medical Research), UMR 1260, Regenerative Nanomedicine, Faculty of Pharmacy, University of Strasbourg, 67000 Strasbourg, France

^2^Hôpitaux Universitaires de Strasbourg, Service de Cardiologie, 67000 Strasbourg, France

** Co-last authors*

**Table S1.** Clinical characteristics of coronary artery disease (CAD) patients.

| Patient | Age | Sex | BMI | Hypertension | Dyslipidemia | Diabetes | Smoking | Family  History of CAD |
| --- | --- | --- | --- | --- | --- | --- | --- | --- |
| CAD1 | 83 | M | 12.8 | + | - | - | - | - |
| CAD2 | 81 | M | 24.2 | - | + | - | - | - |
| CAD3 | 58 | M | 20.3 | + | + | - | + | + |
| CAD4 | 70 | M | 25.5 | + | - | - | + | - |
| CAD5 | 77 | M | 30.3 | + | - | - | - | - |
| CAD6 | 61 | F | 25.4 | - | + | - | + | + |
| CAD7 | 67 | M | 29.6 | + | + | - | + | + |
| CAD8 | 79 | M | 26.2 | + | - | - | - | + |
| CAD9 | 66 | M | 24.5 | + | + | - | + | + |
| CAD10 | 71 | M | 28.1 | + | + | + | + | + |
| CAD11 | 59 | M | 28.7 | + | + | - | - | - |
| CAD12 | 75 | M | 30.9 | + | - | - | + | - |
| CAD13 | 88 | M | 18.8 | + | + | - | - | - |
| CAD14 | 70 | M | 34.6 | - | - | - | + | + |
| CAD15 | 67 | M | 29.1 | + | - | + | - | - |
| CAD16 | 50 | M | 30.1 | - | + | + | + | - |
| CAD17 | 81 | F | 31.3 | + | - | - | + | + |
| CAD18 | 61 | M | 25.5 | - | - | + | + | + |
| CAD19 | 83 | M | 38.0 | + | - | + | + | - |
| CAD20 | 59 | F | 26.0 | + | + | - | + | + |
| CAD21 | 64 | M | 30.9 | + | + | - | + | - |
| CAD22 | 57 | M | 23.9 | - | - | - | - | - |
| CAD23 | 65 | M | 31.6 | - | - | - | - | - |
| CAD24 | 73 | M | 32.7 | + | + | - | + | - |
| CAD25 | 67 | M | 21.6 | + | + | - | + | + |
| CAD26 | 55 | M | 30.9 | - | - | - | + | - |

CAD, coronary artery disease; F, female; M, male; BMI, Body mass index: body mass (kg)/ [height (m)]^2^.

**Table S2.** Phenotype of circulating MPs in CAD patients. The concentration of circulating MPs captured onto insolubilized antibodies to platelet (GPIb^+^), leukocyte (CD11a^+^) and endothelium (CD31^+^ and CD105^+^) was measured by prothrombinase assay as nM phosphatidylserine equivalent (nM PhtdSer eq).

| Patient | nM PhtdSer eq | | | | |
| --- | --- | --- | --- | --- | --- |
|  | **Total MPs** | **GP1b^+^ MPs** | **CD11a^+^ MPs** | **CD31^+^ MPs** | **CD105^+^ MPs** |
| CAD1 | 5.3 | 1.8 | 1.8 | 0.2 | 0.3 |
| CAD2 | 6.6 | 2.0 | 1.8 | 0 | 0.7 |
| CAD3 | 4.9 | 2.1 | 1.7 | 0.2 | 0.2 |
| CAD4 | 10 | 3.8 | 1.9 | 0 | 0.4 |
| CAD5 | 3.4 | 1.6 | 1.7 | 0 | 0.2 |
| CAD6 | 4.2 | 0.5 | 2.8 | 0 | 0.3 |
| CAD7 | 4.4 | 1.1 | 2.9 | 0.1 | 0.5 |
| CAD8 | 5.2 | 2.0 | 2.3 | 0 | 0.3 |
| CAD9 | 5.1 | 0.7 | 2.0 | 0 | 0.1 |
| CAD10 | 4.6 | 1.0 | 3.9 | 0.1 | 0.8 |
| CAD11 | 7.4 | 1.4 | 5.3 | 0 | 0.6 |
| CAD12 | 5.2 | 1.8 | 3.4 | 0 | 0.5 |
| CAD13 | 11.5 | 2.9 | 4.1 | 0.1 | 0.6 |
| CAD14 | 5.9 | 2.4 | 4.4 | 0 | 0.5 |
| CAD15 | 6.2 | 2.2 | 3.1 | 0 | 0.5 |
| CAD16 | 3 | 1.4 | 2.7 | 0.3 | 0.5 |
| CAD17 | 6.1 | 2.1 | 2.4 | 0.3 | 1.0 |
| CAD18 | 7.4 | 3.1 | 3.7 | 0.5 | 1.1 |
| CAD19 | 6.5 | 2.2 | 1.9 | 0.6 | 0.6 |
| CAD20 | 4.6 | 2 | 3.1 | 0.3 | 0.5 |
| CAD21 | 6.4 | 2.1 | 3.6 | 0.1 | 0.8 |
| CAD22 | 7.4 | 1.8 | 1.1 | 0 | 0.2 |
| CAD23 | 4.4 | 1.5 | 1.9 | 0 | 0 |
| CAD24 | 10.7 | 4.4 | 1.0 | 0 | 0.2 |
| CAD25 | 5.3 | 1.0 | 1.3 | 0 | 0.1 |
| CAD26 | 8.3 | 5.6 | 1.8 | 0 | 0.2 |
| Mean | 6.2 | 2.1 | 2.6 | 0.1 | 0.5 |
| SEM | 0.4 | 0.2 | 0.2 | 0.0 | 0.1 |

**Table S3.** Primer sequences. Forward and reverse primer sequences of genes used for RT-qPCR showing position and size. 18S, GUSB and HPRT were used as housekeeping genes.

| **Gene** | **NCBI ref seq** | **position** | **Forward** | **position** | **Reverse** | **size** |
| --- | --- | --- | --- | --- | --- | --- |
| 18S | NR_046261 | 165-182 | GCCCTCGGTCGAGTTGTC | 277-260 | CTTGCAGGGCGGTGACAG | 113 |
| GUSB | NM_001123121 | 297-316 | GGTGTGGTATGAACGGGAGG | 409-390 | CATTCACCCACACAATGGCG | 113 |
| HPRT | NM_001032376 | 112-131 | CCCAGCGTCGTGATTAGTGA | 247-229 | GCCGTTCAGTCCTGTCCATA | 130 |
| TP53 | XM_013977858.2 | 492-510 | CACCCTTGTGCCTCACTCC | 619-600 | AGAAGATCAGCCGGCGTTTG | 128 |
| CDKN1A bis | NM_213824.3 | 454-473 | TCCTGCATTCTGGAACAGCC | 513-494 | GCTTATTGAGGGCAGGGGAG | 60 |

**A	Short-term (30 min)**

**B	Long-term (24 h)**

**VAS-2870**

**(1 𝛍M)**

**N-acetyl cysteine**

**(1 mM)**

**Myxothiazol (0.5 𝛍M)**

**+ KCN (1 𝛍M)**

**+ Rotenone (1 𝛍M)**

**Control**

**Indomethacin**

**(30 𝛍M)**

**Ang II (100 nM, 30 min)**

**Losartan**

**(1 𝛍M)**

**VAS-2870**

**(1 𝛍M)**

**N-acetyl cysteine**

**(1 mM)**

**Myxothiazol (0.5 𝛍M)**

**+ KCN (1 𝛍M)**

**+ Rotenone (1 𝛍M)**

**Control**

**Indomethacin**

**(30 𝛍M)**

**Ang II (100 nM, 24 h)**

**Losartan**

**(1 𝛍M)**

**Figure S1.** The sustained Ang II-induced formation of ROS in ECs involves NADPH oxidase, cyclooxygenases and the mitochondrial respiratory chain. ECs are incubated with either (A, B) N-acetyl cysteine (NAC, an antioxidant) for 2 h, VAS-2870 (VAS, a NADPH oxidase inhibitor), indomethacin (INDO, a cyclooxygenase inhibitor), myxothiazol + KCN + rotenone (MKR, mitochondrial respiratory chain inhibitors) or losartan (LOS, an AT1R antagonist) for 30 min before the addition of Ang II for either 30 min or 24 h and the subsequent determination of dihydroethidium staining by confocal microscope. Results are shown as representative micrography of dihydroethidium staining (left panels) and corresponding cumulative data (right panels). Data are expressed as mean ± SEM of n=3. ^*^*P* < 0.05 vs control and ^#^*P* < 0.05 vs Ang II.

**A**

**B**

**SGLT1**

**β-tubulin**

**SGLT2**

**β-tubulin**

**eNOS**

**β-tubulin**

**VCAM-1**

**β-tubulin**

**C**

**D**

**Figure S2.** Circulating MPs from patients with CAD up-regulate SGLT1, SGLT2, and VCAM-1 and down-regulate eNOS in ECs. (A-D) ECs are exposed to CAD-MPs (10 nM PhtdSer eq) from 10 different CAD patients. Thereafter, the expression level of target proteins is assessed by Western blot analysis. Results are shown as representative immunoblots (upper panels) and corresponding cumulative data (lower panels). Data are expressed as mean ± SEM of n=3. ^*^*P* < 0.05 vs control.
